# Supplementary material for: Under nutrition and associated factors among adolescent girls attending school in the rural and urban districts of Debark, Northwest Ethiopia: A community-based comparative cross-sectional study
Source: PLoS One. 2021 Aug 16;16(8):e0254166. doi: 10.1371/journal.pone.0254166 (PMC8366968; doi:10.1371/journal.pone.0254166)
Supplement: S1 Questionnaire — (DOCX) [file pone.0254166.s001.docx]

**English version Questionnaire**

Data collector Signature ---------------------------------------------------

Date -----------------------------------------------------

Code number of the Questionnaire ___________Name of the school_____________

| 1.**socio demographic/economics characteristics** | | | | |
| --- | --- | --- | --- | --- |
| 101 | | Age | …………………………in year |  |
| 102 | | Level of education | ………………………… in grade |  |
| 103 | | Religion | 1. Orthodox 2. Muslim  3. Protestant 4.Others (Specify)………. |  |
| 104 | | Marital status | 1. Married  2. Single  3. Divorced  4. Widowed  5. Separated |  |
| 105 | | Educational status of the father | 1.Unable to read and write  2.read and write  3.Primary school(1-8)  4.Secondary school(9-12)  5.College and above |  |
| 106 | | Occupation of father | 1.Government Employee  2.Farmer  3.Daily Labourer  4.Merchant  5. Others (Specify)……………… |  |
| 107 | | Education status of the mother | 1.Unable To Read And Write  2.Read And Write  3.Primary School(1-8)  4.Secondary School (9-12)  5.College And Above |  |
| 108 | | Occupation of mother | 1.Housewife  2.Government Employee  3.farmer  4.Daily Labour  5.Merchant  6. Other (Specify)…… |  |
| 109 | | Total family size | ......................in number |  |
| **Part 2: Household environment and hygiene characteristics** | | | | |
| 201 | | Source of drinking water? | 1.tap water in the house  2.Protected spring  3.Unprotected spring  4.Protected well  5.unprotected well  6.river  7.Other specify |  |
| 202 | | Water treatment methods? | 1.boiling  2.adding chlorine  3.strain through a cloth  4.using sand  5.using filtering materials  6. Others.....  7.Don’t know |  |
| 203 | | How long does it take to fetch water? You go and come back | In minutes__________  If the tap in the compound put- 0 |  |
| 204 | | Availability of latrine? | 1. Yes 2. No |  |
| 205 | | Do you wash your hands after visited Toilet? | 1. No 2.Yes |  |
| 206 | | Availability of west disposal/garbage | 1. Yes 2. No |  |
| **Part 3: Health and nutrition information characteristics** | | | | |
| 301 | | How often do you attend television related to health and nutrition in a week? | 1.I do not watching  2.less than  3.at least once  4.very often |  |
| 302 | | How often do you attend radio related to health and nutrition? | 1.I do not listen  2.less than ones a week  3.at least ones a week  4.very often |  |
| 303 | How often do you attend a newspaper, Magazine related to health and nutrition? | | 1.I do not read  2.less than ones a week  3.at least ones a week  4.very often |  |
| **Part 4 health related factors** | | | | |
| 401 | Have you begun menstruation? | | 1.yes  2. No | If 2 skip to 501 |
| 402 | At what age did you see your first menstruation | | ......................in year  I do not remember 1 |  |
| 403 | Do you have diarrhea in the last two Weeks? | | 1. Yes 2. No |  |

| **Part 5:Food Frequency and House Hold food security assessment tool** | | | |
| --- | --- | --- | --- |
| 501 | In the previous day  (Yesterday) day and night (24hr) How many times did you eat? (with snack) | .............in number |  |
| 502 | In the past four weeks, did you worry that your household would not have enough food? | No...........................0  Yes..........................1 |  |
| 503 | How often did this happen? | Never occurred in the past four weeks………0  Rarely (once or twice in the past four weeks)..........................1  Sometimes (three to ten times in the past four weeks)............2  Often (more than ten times in the past four weeks)....................3 |  |
| 504 | In the past four weeks, were you or any household member not able to eat the kinds of foods you preferred because of a lack of resources? | No..................................0  Yes.................................1 |  |
| 505 | How often did this happen? | Never occurred in the past for weeks………0  Rarely (once or twice in the past four weeks)..........................1  Sometimes (three to ten times in the past four weeks)............2  Often (more than ten times in the past four weeks)....................3 |  |
| 506 | In the past four weeks, did you or any household member have to eat a limited variety of foods due to a lack of resources? | No..................................0  Yes.................................1 |  |
| 507 | How often did this happen? | Never occurred in the past for weeks………0  Rarely (once or twice in the past four weeks)..........................1  Sometimes (three to ten times in the past four weeks)............2  Often (more than ten times in the past four weeks)....................3 |  |
| 508 | In the past four weeks, did you or any household member have to eat some foods that you really did not want to eat because of lack of resources to obtain other types of food? | No..................................0  Yes.................................1 |  |
| 509 | How often did this happen? | Never occurred in the past for weeks………0  Rarely (once or twice in the past four weeks)..........................1  Sometimes (three to ten times in the past four weeks)............2  Often (more than ten times in the past four weeks)....................3 |  |
| 510 | In the past four weeks, did you or any household member have to eat a smaller meal than you felt you needed because there was not enough food? | No..................................0  Yes.................................1 |  |
| 511 | How often did this happen? | Never occurred in the past for weeks………0  Rarely (once or twice in the past four weeks)..........................1  Sometimes (three to ten times in the past four weeks)............2  Often (more than ten times in the past four weeks)....................3 |  |
| 512 | In the past four weeks, did you or any other household member have to eat fewer meals in a day because there was not enough food? | No..................................0  Yes.................................1 |  |
| 513 | How often did this happen? | Never occurred in the past for weeks………0  Rarely (once or twice in the past four weeks)..........................1  Sometimes (three to ten times in the past four weeks)............2  Often (more than ten times in the past four weeks)....................3 |  |
| 514 | In the past four weeks, was there ever no food to eat of any kind in your household because of lack of resources to get food? | No..................................0  Yes.................................1 |  |
| 515 | How often did this happen? | Never occurred in the past for weeks………0  Rarely (once or twice in the past four weeks)..........................1  Sometimes (three to ten times in the past four weeks)............2  Often (more than ten times in the past four weeks)....................3 |  |
| 516 | In the past four weeks, did you or any household member go to sleep at night hungry because there was not enough food? | No..................................0  Yes.................................1 |  |
| 517 | How often did this happen? | Never occurred in the past for weeks………0  Rarely (once or twice in the past four weeks)..........................1  Sometimes (three to ten times in the past four weeks)............2  Often (more than ten times in the past four weeks)....................3 |  |
| 518 | In the past four weeks, did you or any household member go a whole day and night without eating anything because there was not enough food? | No..................................0  Yes.................................1 |  |
| 519 | How often did this happen? | Never occurred in the past for weeks………0  Rarely (once or twice in the past four weeks)..........................1  Sometimes (three to ten times in the past four weeks)............2  Often (more than ten times in the past four weeks)....................3 |  |
| **Part 6:Dietary diversity score tool with 24-hour dietary recall**  **Read the list of foods please put one in the box if the food in question was eaten, and please put two in the box if the food was not eaten.** | | | |
|  | **Food category** |  | **Consumed**  **Yes=1**  **No=0** |
| 601 | Any food which is made from Grains, white roots and tubers, and plantains | Breads, rice, stiff porridges of maize, sorghum/millet, pasta, potatoes, teff, wheat, rice, barley, maize, and oats. | 1 0 |
| 602 | Any food which is made from Pulses (beans, peas and lentils) | bean, pea, lentil | 1 0 |
| 603 | Any food which is made from Nuts and seeds | sesame, flax, sunflower, and nuts, nigger | 1 0 |
| 604 | Any food which is made from Dairy and dairy products | Milk, soft and hard cheeses and yoghurt | 1 0 |
| 605 | Any food which is made from Meat, poultry and fish | Meats, organ meats, poultry ,fish beef | 1 0 |
| 606 | Any food which is made from Eggs | Eggs from any type of bird | 1 0 |
| 607 | Any food which is made from Dark green leafy vegetables | Chili, cabbage, spinach salad, | 1 0 |
| 608 | Any food which is made from Other vitamin A-rich fruits and vegetables | Potato, carrot, pumpkin ,pepper, and deep yellow- or orange | 1 0 |
| 609 | Any food which is made from Other vegetables | Onion, Tomato, and mushroom | 1 0 |
| 610 | Any food which is made from Other fruits | Orange, Banana, Avocado, watermelon, Apple, and Lemon | 1 0 |

| **Part 7: household wealth index related characteristics** | | | |
| --- | --- | --- | --- |
| Code | Questions | Response | skip |
| 701 | Owner ship of the house | 1. Private 2. Rented from individual 3. kebele 4. Others (specify)___________ |  |
| 702 | Number of rooms | ____________in number |  |
| 703 | Main material of the dwelling floor | 1. Earth / Sand 2. Dung 3. Cement 4. ceramic 5. Others (specify)__________ |  |
| 704 | Main material of the roof | 1. Iron corrugated sheet 2. Wood 3. Thatch 4. Others (specify)__________ |  |
| 705 | Main material of the exterior walls | 1. Stone with mud 2. Wood with mud 3. Stone with cement 4. Others (specify)__________ |  |
| 706 | Type of fuel mainly used for household cooking | 1. Electricity 2. Charcoal 3. Kerosene 4. Wood 5. Animal dung 6. Others(specify)___________ |  |
| 707 | Is the cooking usually done in the house, in a separate building, or outdoors? | 1. In a separate room used as kitchen 2. Elsewhere in the house 3. Outdoors 4. Other (specify)___________ |  |
| 708 | Does any member of the household own any land that can be used for agriculture? | 1. Yes 2. No | 711 |
| 709 | Ownership of the farm land (if the household doesn’t have one of the two option, use 999) | 1. Own, in hectares 2. Rent, in hectares |  |
| 710 | Annual total agricultural products(includes all items) | ______________quintal |  |
| 711 | Monthly income | ………………… in birr |  |
| 712 | Does your household have   1. Electricity? 2. A Radio? 3. A Television? 4. A Non-mobile telephone? 5. A Refrigerator? 6. Table? 7. Chair? 8. A bed with cotton/spring mattress | \| Yes \| No \| \| --- \| --- \| \| 1 \| 0 \| \| 1 \| 0 \| \| 1 \| 0 \| \| 1 \| 0 \| \| 1 \| 0 \| \| 1 \| 0 \| \| 1 \| 0 \| \|  \|  \| \| 1 \| 0 \| |  |
| 713 | Does any member of your household own   1. A watch? 2. A mobile phone? 3. A bicycle? 4. A Bajaj? 5. Animal drawn cart? 6. Car? | \| Yes \| No \| \| --- \| --- \| \| 1 \| 0 \| \| 1 \| 0 \| \| 1 \| 0 \| \| 1 \| 0 \| \| 1 \| 0 \| \| 1 \| 0 \| |  |
| 714 | Does this household own any livestock, herds, other farm animals, or poultry? | 1. Yes 2. No | 716 |
| 715 | How many of the following animals does the household have? |  |  |
|  | 1. Cattle, milk cows, bulls? | _________in number |  |
|  | 1. Horses, Donkeys, or mules? | _________in number |  |
|  | 1. Goats? | _________in number |  |
|  | 1. Sheep? | _________in number |  |
|  | 1. Chickens? | _________in number |  |
|  | 1. Beehives? | _________in number |  |
| 716 | Does any member of this household have a bank account? | 1. Yes 2. No |  |

| **8.Anthropometric measurements** | | | |
| --- | --- | --- | --- |
| 801 | Height | .....................................cm |  |
| 802 | Weight | ........................................kg |  |
