# Supplementary material for: Under nutrition and associated factors among adolescent girls attending school in the rural and urban districts of Debark, Northwest Ethiopia: A community-based comparative cross-sectional study
Source: PLoS One. 2021 Aug 16;16(8):e0254166. doi: 10.1371/journal.pone.0254166 (PMC8366968; doi:10.1371/journal.pone.0254166)
Supplement: S2 Questionnaire — (DOCX) [file pone.0254166.s002.docx]

**Amharic version** **Questionnaire**

የተቆጣጣሪው ሙሉ ስም------------------------------------------------ፊርማ---------------------ቀን---------

የመጠይቁ ኮድ ----------------------------------------------------------የት/ቤት ስም --------------------------

| ተ. ቁ | ጥያቄዎች | | አማራጭ መልሶች (መልሱን ያክቡ) | | | ወደ ተራ ቁጥር-ይዝለሉ | | | |
| --- | --- | --- | --- | --- | --- | --- | --- | --- | --- |
| ክፍል 1. ማህበራዊ፤ኢኮኖሚያዊ እና ስነ-ህዝባዊ መረጃን በተመለከተ ዳሰሳ | | | | | | | | | |
| 101 | ዕድሜ | | …………/…………/……በአመት | | |  | | | |
| 102 | የትምህርት ደረጃ | | ………………………...በክፍል | | |  | | | |
| 103 | ሃይማኖት | | 1. ኦድቶዶክስ 3. ፕሮቴስታንት  2. ሙስሊም 4. ሌላ ካለ(ይገለጥ) | | |  | | | |
| 104 | የጋብቻ ሁኔታ | | 1. ያገባች 4. ባል የሞተባት  2. ያላገባች 5. የተለያየች  3. የፈታች | | |  | | | |
| 105 | የአባት የትምህርት ደረጃ | | 1. ማንበብና መጻፍ የማይችል  2. ማንበብና መጻፍ የሚችል  3. የመጀመሪያ ደረጃ የጨረሰ(1-8)  4. ሁለተነኛ ደረጃ የጨረሰ(9-12)  5. ኮሌጅና ከዚያ በላይ | | |  | | | |
| 106 | የአባት የስራ ሁኔታ | | 1. የመንግሰት ሰራተኛ 4. ነጋዴ  2. አርሶ አደር 5. ሌላ ካለ ይገለጥ...  3. የቀን ሰራተኛ | | |  | | | |
| 107 | የእናት የትምህርት ደረጃ | | 1. ማንበብና መጻፍ የማትችል  2. ማንበብና መጻፍ የምትችል  3. የመጀመሪያ ደረጃ የጨረሰች(1-8)  4. ሁለተነኛ ደረጃ የጨረሰች(9-12)  5. ኮሌጅና ከዚያ በላይ | | |  | | | |
| 108 | የእናት የስራ ሁኔታ | | 1. የመንግሰት ሰራተኛ 4. የቀን ሰራተኛ  2. የቤት እመቤት 5. ነጋዴ  3. አርሶ አደር 6. ሌላ ካለ ይገለጥ... | | |  | | | |
| 109 | የቤተሰብ ቁጥር ብዛት | | ..........................በቁጥር | | |  | | | |
| ክፍል 2. የቤተሰብና የአካባቢን ንጽህና በተመለከተ ዳሰሳ | | | | | | | | | |
| 201 | የቤተሰቡ ዋና የመጠጥ ውኃ ምንጭ ምንድን ነው? | | 1.መኖሪያ ቤት ውስጥ ያለ ቧንቧ ውሃ  2. ንጽኅናው የተጠበቀ የጉድጓድ ውኃ  3. ንጽኅናው ያልተጠበቀ የጉድጓድ ውኃ  4. ንጽኅናው የተጠበቀ የምንጭ ውሃ  5. ንጽኅናው ያልተጠበቀ የምንጭ ውሃ  6. የወንዝ/መስኖ ውሃ  7. ሌላ ካለ ይገለጥ... | | | | |  | |
| 202 | የውሃውን ንፅህና ለመጠበቅ የሚጠቀሙት ዘዴ ምንድ ነው? (ካንድ በላይ መልስ መስጠት ይቻላል) | | 1.ውሃውን ማፍላት  2. ክሎሪን በመጨመር  3. ውሃ ማጥለያ በመጠቀም(ሸክላ፤አሸዋ)  4. ቆሻሻው እንዲዘቅጥ በማድረግ  5. የውሃ ማጣሪያ መሳሪያዎችን በመጠቀም  6. ሌላ......  7. አይታወቅም | | | | |  | |
| 203 | በአብዛኛው ውኃ ቀድቶ ለመመለስ ምን ያሕል ጊዜ ይፈጅበዎታል? | | .........በደቂቃ  ግቢ ውስጥ ከሆነ- 0 | | | | |  | |
| 204 | መፀዳጃ ቤት አለ? | | 1.አለ 0. የለም | | | | |  | |
| 205 | ከመፀዳጃ ቤት መልስ እጀዎትን ይታጠባሉ? | | 1.የለም  2. አዎ | | | | |  | |
| 206 | ቆሻሻ ማሰወገጃ አለ? | | 1. አዎ 0. የለም | | | | |  | |
| ክፍል 3. የጤና እና የስነ-ምግብ የመረጃ ምንጮችን በተመለከተ ዳሰሳ | | | | | | | | | |
| 301 | ለምን ያህል ጊዜ የጤና እና የስነ-ምግብ የመረጃ ምንጮችን በተመለከተ ቴሌቪዝን ይመለከታሉ? | | | 1. አላይም  2. በሳምንት ከአንድ ግዜ በታች  3. ቢያንስ በሳምንት ውስጥ አንድ ግዜ  4. በተደጋጋሚ | | |  | | |
| 302 | ለምን ያህል ጊዜ ራዲዮ ያደምጣሉ? | | | 1. አላዳምጥም  2. በሳምንት ከአንድ ግዜ በታች  3. ቢያንስ በሳምንት ውስጥ አንድ ግዜ  4. በተደጋጋሚ | | |  | | |
| 303 | ለምን ያህል ጊዜ መጽሔት፣ ጋዜጣ ያነባሉ? | | | 1. አላነብም  2. በሳምንት ከአንድ ግዜ በታች  3. ቢያንስ በሳምንት ውስጥ አንድ ግዜ  4. በተደጋጋሚ | | |  | | |
| ፍል 4. የጤና ሁናታን በተመለከተ ዳሰሳ | | | | | | | | | |
| 401 | የወር አበባ ማየት ጀምረሻል? | | | 1. አዎ  0. የለም | | | የለም ካሉ ወደ ጥያቄ 403 ይቀጥሉ | | |
| 402 | የወር አበባ ማየት የጀመርሽው በስንት አመትሽ ነው? | | | ..............አመት | | |  | | |
| 403 | ባለፈው ሁለት ሳምንታት ውስጥ በተቅማጥ በሽታ ታመው ነበር? | | | 1. አዎ  0. የለም | | |  | | |
| **ክፍል 5፡ የምግብ ድግግሞሽ ዳሰሳ** **እና የምግብ ዋስትናን በተመለክተ የተዘጋጀ**  ከጥያቄ 501- 519 ድረስ ያሉት ጥያቄዎች **ባለፉት 4 ሳምንታት** ቤተሰቡ ወይም ከቤተሰብ አባላት መካከል አንዱም ቢሆን በችግር ምክንያት ሊያጋጥማቸው ስለሚችል የምግብ አቅርቦት እና የአመጋገብ ሁኔታን የሚጠይቁ ጥያቄዎች ናቸው፡፡ | | | | | | | | |  |
| 501 | | በትናትናው እለት ጥዋትም ሆነ ማታ  (በ 24 ሰአት ውስጥ) ምን ያህል ጊዜ ተመግበዋል? (መክሰስን ጨምሮ) | | | .......................ጊዜ በቁጥር | |  | |  |
| 502 | | ቤተሰቡ በቂ ምግብ አያገኝ ይሆናል ብለው ተጨንቀው ያውቃሉ? | | | የለም--------------------------0  አዎ----------------------------1 | |  | |  |
| 503 | | ቤተሰቡ በቂ ምግብ አያገኝ ይሆናል ብለው ተጨንቀው ከሆነ ስንት ጊዜ ነበር? | | | በ4 ሳምንት ውስጥ በጭራሽ አልተከሰተም…………0  በጣም ትንሽ ጊዜ (በ4 ሳምንት 1-2 ጊዜ)----1  አንዳንድ ጊዜ (በ4 ሳምንት 3-10 ጊዜ)--------2  ብዙ ጊዜ (በ4 ሳምንት ከ10 ጊዜ በላይ)------3 | |  | |  |
| 504 | | ቤተሰቡ ወይም ከቤተሰብ አባላት መካከል አንዱም ቢሆን መመገብ የሚፈልገውን ምግብ ማግኝት ያልቻለበት ሁኔታ ነበር? | | | የለም--------------------------0  አዎ----------------------------1 | |  | |  |
| 505 | | የሚፈለገው አይነት ምግብ ያልተገኘው ስንት ጊዜ ነበር**?** | | | በ4 ሳምንት ውስጥ በጭራሽ አልተከሰተም…………0  በጣም ትንሽ ጊዜ (በ4 ሳምንት 1-2 ጊዜ)----1  አንዳንድ ጊዜ (በ4 ሳምንት 3-10 ጊዜ)--------2  ብዙ ጊዜ (በ4 ሳምንት ከ10 ጊዜ በላይ)------3 | |  | |  |
| 506 | | የቤተሰቡ አባላት የተወሰነ ምግብ አይነት ብቻ ለመብላት የተገደዱበት ሁኔታ ነበር? | | | የለም--------------------------0  አዎ-----------------------------1 | |  | |  |
| 507 | | የቤተሰቡ አባላት የተወሰነ ምግብ አይነት ብቻ ለመብላት የተገደዱት ስንት ጊዜ ነበር? | | | በ4 ሳምንት ውስጥ በጭራሽ አልተከሰተም…………0  በጣም ትንሽ ጊዜ (በ4 ሳምንት 1-2 ጊዜ)----1  አንዳንድ ጊዜ (በ4 ሳምንት 3-10 ጊዜ)--------2  ብዙ ጊዜ (በ4 ሳምንት ከ10 ጊዜ በላይ)------3 | |  | |  |
| 508 | | ቤተሰቡ ወይም ከቤተሰብ አባላት መካከል አንዱም ቢሆን መመገብ የማይፈልገውን ምግብ እንዲበላ የተገደደበት ሁኔታ ነበር? | | | የለም--------------------------0  አዎ-----------------------------1 | |  | |  |
| 509 | | ቤተሰቡ ወይም ከቤተሰብ አባላት መካከል የማይፈልገውን አይነት ምግብ እንዲበላ የተገደደው ስንት ጊዜ ነበር? | | | በ4 ሳምንት ውስጥ በጭራሽ አልተከሰተም…………0  በጣም ትንሽ ጊዜ (በ4 ሳምንት 1-2 ጊዜ)----1  አንዳንድ ጊዜ (በ4 ሳምንት 3-10 ጊዜ)--------2  ብዙ ጊዜ (በ4 ሳምንት ከ10 ጊዜ በላይ)------3 | |  | |  |
| 510 | | ቤተሰቡ ወይም ከቤተሰብ አባላት መካከል አንዱም ቢሆን ከወትሮው በመጠኑ ያነስ ምግብ እንዲበላ የተገደደበት ሁኔታ ነበር? | | | የለም--------------------------0  አዎ----------------------------1 | |  | |  |
| 511 | | ቤተሰቡ ወይም ከቤተሰብ አባላት መካከል አንዱም ቢሆን ከወትሮው በመጠኑ ያነስ ምግብ እንዲበላ የተገደደው ስንት ጊዜ ነበር? | | | በ4 ሳምንት ውስጥ በጭራሽ አልተከሰተም…………0  በጣም ትንሽ ጊዜ (በ4 ሳምንት 1-2 ጊዜ)----1  አንዳንድ ጊዜ (በ4 ሳምንት 3-10 ጊዜ)--------2  ብዙ ጊዜ (በ4 ሳምንት ከ10 ጊዜ በላይ)------3 | |  | |  |
| 512 | | ቤተሰቡ ወይም ከቤተሰብ አባላት መካከል አንዱም ቢሆን ምግብ ከወትሮው በቀን ከሚበላው ያነስ ጊዜ እንዲበላ የተገደደበት ሁኔታ ነበር? | | | የለም--------------------------0  አዎ-----------------------------1 | |  | |  |
| 513 | | ቤተሰቡ ወይም ከቤተሰብ አባላት መካከል አንዱም ቢሆን ምግብ ከወትሮው በቀን ከሚበሊው ያነስ ጊዜ እንዲበላ የተገደደው ስንት ጊዜ ነበር? | | | በ4 ሳምንት ውስጥ በጭራሽ አልተከሰተም…………0  በጣም ትንሽ ጊዜ (በ4 ሳምንት 1-2 ጊዜ)----1  አንዳንድ ጊዜ (በ4 ሳምንት 3-10 ጊዜ)--------2  ብዙ ጊዜ (በ4 ሳምንት ከ10 ጊዜ በላይ)------3 | |  | |  |
| 514 | | በቤት ውስጥ በችግር ምክንያት ምንም አይነት ምግብ የጠፋበት ሁኔታ ነበር? | | | የለም--------------------------0  አዎ----------------------------1 | |  | |  |
| 515 | | በቤት ውስጥ ምንም አይነት ምግብ የጠፋው ስንት ጊዜ ነበር? | | | በ4 ሳምንት ውስጥ በጭራሽ አልተከሰተም…………0  በጣም ትንሽ ጊዜ (በ4 ሳምንት 1-2 ጊዜ)----1  አንዳንድ ጊዜ (በ4 ሳምንት 3-10 ጊዜ)--------2  ብዙ ጊዜ (በ4 ሳምንት ከ10 ጊዜ በላይ)------3 | |  | |  |
| 516 | | ቤተሰቡ ወይም ከቤተሰብ አባላት መካከል አንዱም ቢሆን ምግብ ሳይበላ ተርቦ ያደረበት ጊዜ ነበር? | | | የለም--------------------------0  አዎ----------------------------1 | |  | |  |
| 517 | | የምግብ እጥረት በመኖሩ ምክንያት ምግብ ሳይበላ ተርቦ ያደረው ስንት ጊዜ ነበር? | | | በ4 ሳምንት ውስጥ በጭራሽ አልተከሰተም…………0  በጣም ትንሽ ጊዜ (በ4 ሳምንት 1-2 ጊዜ)----1  አንዳንድ ጊዜ (በ4 ሳምንት 3-10 ጊዜ)--------2  ብዙ ጊዜ (በ4 ሳምንት ከ10 ጊዜ በላይ)------3 | |  | |  |
| 518 | | ቤተሰቡ ወይም ከቤተሰብ አባላት መካከል አንዱም ቢሆን ምንም ምግብ ሳይበላ ውሎ ያደረበት ጊዜ ነበር? | | | የለም-------------------------0  አዎ---------------------------1 | |  | |  |
| 519 | | ምግብ ሳይበላ ውሎ ያደረው ስንት ጊዜ ነበር? | | | በ4 ሳምንት ውስጥ በጭራሽ አልተከሰተም…………0  በጣም ትንሽ ጊዜ (በ4 ሳምንት 1-2 ጊዜ)----1  አንዳንድ ጊዜ (በ4 ሳምንት 3-10 ጊዜ)--------2  ብዙ ጊዜ (በ4 ሳምንት ከ10 ጊዜ በላይ)------3 | |  | |  |

| 6. በምግብ ጊዜ የምግብ ስብጥር ሁኔታ የሚዳስስ  ትናንትናጠዋትከተመገቡትምግብበመነሳትበትናንትናውዕለትቀንምሆነማታ(ፀሐይከወጣችጀመወሮለ24 ሰዓት) በተናጠል ወይም በጥምር የወሰዷቸውን የምግብ/የመጠጥ አይነቶች ምንምን እንደሆኑ(ከተዘረዘሩት ውስጥአንዱን እና ከዚያ በላይ ከተጠቀሙ 1ን ምንም ካልተጠቀሙ 0) ይግለጹ፡፡ | | | |
| --- | --- | --- | --- |
| ተ. ቁ | ጥያቄዎች | ምሳሌዎች | 1.አዎ0.የለም |
| 601. | ባለፈው 24 ሰአት ውስጥ ቀንም ሆነማታ የእህል ዘር እና ስር ያላቸው ተመግበዋል? | እንጀራ፣ዳቦ፣ቂጣ፣ገንፎ፣ማሽላ፣በቆሎ፣ሩዝ፣ፓስታ፣ስንዴ፣ገብስ፣ዳጉሳ፣፣ድንች፣ቆጮ፣ካዛባ፣ቀይ.ስር፣አጃ | 1 0 |
| 602 | ባለፈው 24 ሰአት ውስጥ ከጥራጥሬ ዘር ተመግበዋል? | ባቄላ፣አተር፣ምስር፣ጋያ፣ምስር | 1 0 |
| 603 | ከቅባትእህልዘርተመግበዋል? | ኦቾሎኒ፣ኑግ፣ተልባ፣ለውዝ፣ሰሊጥናሱፍ እና ሌሎች. ከቅባት እህል ዘሮች | 1 0 |
| 604. | ባለፈው 24 ሰአት ውስጥ ወተትና የወተት ተዋጽኦ ተመግበዋል? | ወተት፣አይብ፣እርጎ፣ሌሎችየወተትተዋጽኦ | 1 0 |
| 605 | ባለፈው 24 ሰአት ውስጥ ስጋ፣ የአካል ክፍል ስጋዎች እና የባህር ውስጥ ምግቦችን ተመግበዋ? | የበሬ፣የበግ፣የፍየል፣የዶሮ፣ጉበት፣ኩላሊት፣ልብ፣ወይምየሆድዕቃጨምሮአካልክፍልስጋዎች፣አሣ ሌላ | 1 0 |
| 606 | ባለፈው 24 ሰአት ውስጥ እቁላል ተመግበዋል? | የዶሮ፣የዝግራ፣ሌላ... | 1 0 |
| 607. | ባለፈው 24 ሰአት ውስጥ ደማቅ አረንጋዴ ቅጠል ያላቸው አትክልቶች ተመግበዋል? | ሰላጣ፣ቆስጣ፣ጥቅልጎመን፣ጎመን | 1 0 |
| 608 | ባለፈው 24 ሰአት ውስጥ በቫይታሚን ኤ የበለጸጉ ፍራፍሬዎችና አትክልቶች ተመግበዋል? | ዱባ፣ካሮት፣ስካርድንች፣ድንች፣በርበሬ፣ደማቅቢጫብረቱካን፤ፓፓያ፤ | 1 0 |
| 609 | ባለፈው 24 ሰአት ውስጥ ሌሎች ከላይ ያልተጠቀሱ አተክልቶች ተመግበዋል? | ቀይሽንኩርት፣ነጭሽንኩርት፣ቲማቲም፣እንጉዳይ.... | 1 0 |
| 610 | ሌሎች ፍራፍዎች | ሙዝ፣አቮካዶ፣አናናስ፣አፕል፣ብርቱካን፣ሎሚ፣ሐብሐብ፣ | 1 0 |

| **7. የቤተሰብ ገቢ እና ንብረት ሁኔታን የተመለከቱ ጥያቄዎች** | | | |
| --- | --- | --- | --- |
| ተ. ቁ | ጥያቄዎች | መልሶች | ምርመራ |
| 701 | የቤት ባለቤትነት | 1.የግል 2. የኪራይ  3.የቀበሌ 4. ሌላ ይግለፁ |  |
| 702 | ቤታችሁ ስንት ክፍል አለው | ____________ በቁጥር |  |
| 703 | የቤታችሁ ወለል ከምን የተሰራ ነው | 1.አፈር/አሸዋ 4. ሴራሚክ  2.በእበትየተለቀለቀ 5. ሌላይግለፁ  3.ሲሚንቶ |  |
| 704 | የቤታችሁ ጣራ ከምን የተሰራ ነው | 1.ከብረት/ቆርቆሮ  2.ከእንጨት  3. የሳር ክዳን  4.ሌላ ይግለፁ |  |
| 705 | የቤቱ የዉጭ ግድግዳ በዋናነት ከምን የተሰራ ነው | 1.ድንጋይ እና ጭቃ  2.ከእንጨት እና ከጭቃ  3.በሲሚንቶ የተገረፈ  4.ሌላ ይግለፁ |  |
| 706 | ቤት ዉስጥ በዋናነት ምግብ ለማብሰል የምትጠቀሙት የሃይልምንጭ | 1. ኤሌክትሪክ 2. ከሰል 3. ጋዝ/ነዳጅ 4. እንጭት 5. ኩበት 6. ሌላይግለፁ |  |
| 707 | ምግብ ማብሰያው አብዛኛውን ጊዜ የሚከናወነው በቤቱ ፣ በተለየ ክፍል ወይም ከቤት ውጭ ነው? | 1. ኩሽና 2. ቤት ውስጥ 3. ከቤት ውጭ 4. ሌላ ይግለፁ |  |
| 708 | ማንኛውም የቤተሰብ አባል ለእርሻ ሊያገለግል የሚችል መሬት አለው | 1. አዎ   0.የለም | 711 |
| 709 | የእርሻ መሬቱ ባለቤትነት የማን ነው | 1. የራሳቸው /በሄክታር ____ 2. የኪራይ/በሄክታር ____ |  |
| 710 | ዓመታዊ ጠቅላላ የእርሻ ምርቶች (ሁሉንም ምርቶች ይጨምራል) | ______________ በኩንታል |  |
| 711 | የወር ገቢ | …………በቁጥር |  |
| 712 | የሚከተሉትነገሮችበቤታችሁይገኛሉ   1. መብራት(ኤሌትሪክ)..... 2. ሬድዮ 3. ቴሌቪዥን 4. የቤት ስልክ... 5. ፍሪጅ 6. ጠረጴዛ 7. ወንበር 8. አልጋከጥጥ/ከስፖንጅ/ከስፕሪንግምንጣፍጋር | \| አዎ የለም \|  \| \| --- \| --- \| \| 1  1 \| 0  0 \| \| 1 \| 0 \| \| 1 \| 0 \| \| 1 \| 0 \| \| 1 \| 0 \| \| 1 \| 0 \| \| 1 \| 0 \| \|  \|  \| \|  \|  \| |  |
| 713 | የሚከተሉትነገሮችቤታችሁዉስጥያለውሰውአለወይ?   1. ተንቀሳቃሽ ስልክ 2. ሞተር ብስክሌት 3. ባጃጅ 4. ጋሪ 5. መኪና | \| አዎ የለም \|  \| \| --- \| --- \| \| 1 \| 0 \| \| 1 \| 0 \| \| 1 \| 0 \| \| 1 \| 0 \| \| 1 \| 0 \| \|  \|  \| |  |
| 714 | ይህ ቤተሰብ ማንኛውም ከብት ፣ ሌሎች እንስሳት ወይም የዶሮ እርባታ አለው | 1. አዎ 2. የለም | 716 |
| 715 | ከሚከተሉት እንስሳት ውስጥ ምን ያህሉ ቤት ውስጥ አሉ? |  |  |
|  | 1. በሬ/ላም | _________ በቁጥር |  |
|  | 1. ፈረስ/አህያ/በቅሎ | _________ በቁጥር |  |
|  | 1. ፍየል | _________ በቁጥር |  |
|  | 1. በግ | _________ በቁጥር |  |
|  | 1. ዶሮ | _________በቁጥር |  |
|  | 1. የንብቀፎ | _________ በቁጥር |  |
| 716 | ከቤተሰቡ አባላት መካከል የባንክ ደብተር ያለው ሰው አለ? | 1.አዎ  0.የለም |  |

| ክፍል 8. መጠነ ልኬት | | | |
| --- | --- | --- | --- |
| 801 | ቁመት | ............................ሴሜ |  |
| 802 | ክብደት | ............................ኪግ |  |
