# Supplementary material for: Under nutrition and associated factors among adolescent girls attending school in the rural and urban districts of Debark, Northwest Ethiopia: A community-based comparative cross-sectional study
Source: PLoS One. 2021 Aug 16;16(8):e0254166. doi: 10.1371/journal.pone.0254166 (PMC8366968; doi:10.1371/journal.pone.0254166)
Supplement: Attachment — Submitted filename: COMMENTS-PLOS ONE.docx [file pone.0254166.s004.docx]

Comments on manuscript entitled” **Undernutrition and Associated Factors among Adolescent Girls Attending School in the rural and urban districts of Debark town, Northwest Ethiopia: A community-based comparative cross-sectional study**”. PONE-D-21-06490

The authors assessed the association between the prevalence of undernutrition and different socio-demographic factors among school going adolescent girls of Debark district, Northwest Ethiopia.

I have the following few comments…

1. In the title, the authors mentioned as Adolescent Girls Attending School in the **rural and urban districts of Debark town.** It is not clear, whether Debark is town or district and whether Debark has rural and urban districts.
2. In abstract, total number of adolescent girls was mentioned as 792 in methodology and 757 in results. Need to clarify the same. Correct the error in spelling of stunting.
3. Page 10, line 3. Delete you in “low resistance to infection due **you** to low immunity”
4. Authors reported in methodology as—Lottery method was used to select 20% of the 51 schools. Thus, accordingly we need 10 schools; however, a total 13 schools were covered. Similarly, authors have not selected the schools proportionately. Authors need to explain whether they selected the number of schools purposively or there is any rationale behind it.
5. Authors selected 396 adolescent girls each from rural and urban areas. Here also authors have not followed the PPS method for selection of adolescent girls.
6. Discussion part is exhaustive. Need to condense the same
